# Radiographic Diagnosis of Hip Laxity in Rottweilers: Interobserver Agreement at Eight- and Twelve-Months of Age

**DOI:** 10.3390/ani13020231

**Published:** 2023-01-08

**Authors:** Masoud Aghapour, Barbara Bockstahler, Sibylle Kneissl, Aldo Vezzoni, Michaela Gumpenberger, Harald Hechinger, Alexander Tichy, Britta Vidoni

**Affiliations:** 1 Small Animal Surgery, Department for Companion Animals and Horses, University of Veterinary Medicine, 1210 Vienna, Austria; 2 Section of Physical Therapy, Small Animal Surgery, Department for Companion Animals and Horses, University of Veterinary Medicine, 1210 Vienna, Austria; 3 Diagnostic Imaging, Department for Companion Animals and Horses, University of Veterinary Medicine, 1210 Vienna, Austria; 4 Clinica Veterinaria Vezzoni S.R.L., 26100 Cremona, Italy; 5 Platform Bioinformatics and Biostatistics, Department for Biomedical Services, University of Veterinary Medicine, 1210 Vienna, Austria

**Keywords:** hip dysplasia, laxity, coxofemoral joint, radiographic diagnosis, interobserver agreement

## Abstract

**Simple Summary:**

Hip laxity is one of the most frequent orthopedic disorders in dogs, and it is one of the main underlying reasons for canine hip dysplasia. Canine hip dysplasia is a progressive disease influenced by inheritance. Veterinarians can take timely preventive/therapeutic measures when hip laxity is diagnosed early, and prevent the participation of dysplastic dogs in breeding programs to reduce the prevalence of the disease. Effective treatment strategies can increase the welfare of dogs by reducing the severity of the disease at older ages. In this study, we aimed to assess the reproducibility of the protocols reported for the radiographic assessment of canine hip dysplasia in 8- and 12-month-old Rottweilers. Five observers investigated eight radiographic parameters at each examination time. According to the results, there were high interobserver agreements at both ages for the measurable parameters, such as the center edge angle (CEA), dorsal acetabular rim slope (DARS), distraction index (DI), and Norberg angle (NA), whereas the observers recorded poor to moderate agreements for the subjective values, such as the grading of the dorsal acetabular rim (GDAR), grading of the degenerative joint disease (GDJD), location of the center of the femoral head (LCFH), and sclerosis of the cranial acetabular rim (SCAR).

**Abstract:**

Hip laxity is one of the predisposing factors of canine hip dysplasia. The early diagnosis of hip laxity allows veterinarians to prevent the participation of dysplastic dogs in breeding programs, which could lower the disease’s prevalence due to its genetic background. Furthermore, it allows them to plan preventive/therapeutic procedures for mild/near-normal hips to reduce the symptoms of the disease at older ages. A reliable screening program must be repeatable and reproducible, and intra- and inter-observer studies can help us to determine the best methods. In this study, we aimed to evaluate the radiographic parameters used for the early diagnosis of hip dysplasia in Rottweilers at 8 and 12 months of age with five observers to assess the interobserver agreements. According to our findings, there were high interobserver agreements at both ages for the quantitative values, such as the center edge angle (CEA), dorsal acetabular rim slope (DARS), distraction index (DI), and Norberg angle (NA), whereas we recorded from poor to moderate agreements for the qualitative values, such as the grading of the dorsal acetabular rim (GDAR), grading of the degenerative joint disease (GDJD), location of the center of the femoral head (LCFH), and sclerosis of the cranial acetabular rim (SCAR).

## 1. Introduction

Canine hip dysplasia (CHD) is one of the most frequent orthopedic diseases in dogs. This multifactorial and progressive disease manifests as hip laxity and osteoarthritis [1,2]. The main cause of CHD is still under study; however, researchers have reported heredity, breed, body size, weight, rapid growth, and hormonal changes to be the predisposing factors [1,2,3,4]. The laxity of the coxofemoral joint and subluxation of the femoral head are the primary signs of CHD, as well as the underlying reasons for osteoarthritis [5,6,7]. The degeneration of the coxofemoral joint capsule and ligament of the head of the femur with ossification disorders of the acetabulum are the main underlying reasons for the hip laxity incidence [5,8]. Pain, lameness, exercise intolerance, a decreased range of motion of the coxofemoral joints, and osteoarthritis are the main symptoms of CHD.

The early detection of CHD is crucial for timely intervention. The importance of the early detection of hip laxity lies in the fact that the earlier the disorder is detected, the better the preventive or therapeutic methods can be planned to minimize the symptoms of the disease and improve the welfare of the dog. Juvenile pubic symphysiodesis (JPS) is one of the preventive methods that can be performed on dogs with mild–moderate hip laxity from 12 to 20 weeks of age [9]. The JPS is related to age, and the best results are expected between 12 and 16 weeks of age [10,11]. Furthermore, double or triple pelvic osteotomy (DPO or TPO, respectively) can be performed on patients with higher grades of hip laxity between 16 and 32 weeks of age [11].

Globally, veterinarians use different diagnostic programs to diagnose CHD. Most of these programs are based on radiographic and clinical examinations, and veterinarians perform them on adult dogs (from a minimum of 12 months of age). The FCI (Fédération Cynologique Internationale) scoring method is used in many European countries, Asia, Africa, and South America for dogs from a minimum of 12 months of age (18 months for large and giant breeds), which is based on the measurement of the Norberg angle (NA), the evaluation of the shape of the acetabulum, signs of degenerative joint disease (DJD), and the subluxation angle (SA) [12]. The OFA (Orthopedic Foundation for Animals) scoring program is used in the United States and Canada, and it consists of the measurement of the SA and the existence of DJD in dogs from 24 months of age [13]. The BVA/KC (British Veterinary Association/Kennel Club) scoring program is used in Britain, Ireland, Australia, and New Zealand, and it is based on the assessment of the NA, SA, and signs of DJD in dogs from 12 months of age [14,15].

Despite previous screening programs that were applicable to adult dogs (at least from 1 or 2 years of age), the PennHIP (Pennsylvania Hip Improvement Program) screening method is an early diagnostic program that can be performed on dogs from 4 months of age [16]. This program is based on the evaluation of the DJD, the congruity of the femoral head and acetabulum, and the measurement of the laxity of the femoral head through the calculation of the distraction index (DI). The DI is calculated on distraction-view radiographs, and it is the ratio of the distance between the centers of the acetabulum and femoral head and the radius of the head of the femur [16]. The DI is a number between 0 (sound hip) and 1 (severe laxity). The possibility of the CHD incidence in dogs with a DI ≤ 0.3 is low, whereas dogs with a DI ≥ 0.6 are at an increased risk of hip dysplasia [17].

In addition to radiography, which is a primary diagnostic method for the evaluation of the hip joints, veterinarians also frequently use computed tomography (CT) to assess the coxofemoral joints. The possibility of a three-dimensional evaluation of the acetabulum and femur in this method is one of the prominent advantages. Different researchers have used CT to assess the skeletal structure of the coxofemoral joints, predict the occurrence of osteoarthritis at older ages, and measure the hip laxity in dogs [18,19,20]. Despite the high cost and low availability of CT in veterinary clinics, veterinarians are increasingly using this method, and there will likely be more studies in which the researchers use this method in the future.

The aforementioned radiographic programs are the most popular and frequently used diagnostic protocols worldwide; however, in some cases, veterinarians might utilize a combination of different methods, or even other methods, such as the measurement of the subluxation index (SI), for the diagnosis of CHD [21]. These systems are not only relevant for the diagnosis and treatment of CHD, but they also help veterinarians to exclude dogs with severe grades of CHD from breeding programs [3,22]. Recently, researchers have confirmed that long-term selection strategies could reduce the prevalence of CHD in some breeds [23,24]. Despite the fact that genetics is important in this disease, the final score does not only reflect the inheritance potential of CHD in the next generations because environmental factors also play a role in the incidence of this disease [1,2].

Despite all these screening programs, the early detection of CHD is still challenging. Developing new early diagnostic methods is not only important in veterinary medicine, but it might also be applicable to human medicine, as CHD is an experimental model for the evaluation of hip dysplasia and osteoarthritis in human medicine [25,26]. Together with attempts to develop different molecular and paraclinical diagnostic methods to identify the adequate biomarkers for the early diagnosis of CHD [25,27], efforts to develop sensitive clinical methods, such as radiography, are still in progress [28]. The major challenges in the early diagnosis of CHD are the lack of information on the pathophysiology of the disease and its progressive and irreversible nature [25].

Given that many of the evaluated values in radiographic screening programs are qualitative, the interpretation of the findings might vary between observers. Thus, having standard protocols with high interobserver agreements might help to reduce the scoring bias and increase the homogeneity of the results. In our previous study, we showed high and poor interobserver correlations for the quantitative and qualitative values, respectively, in Rottweilers at 4 months of age [29]. Considering that many of the symptoms of hip laxity or signs of DJD may not be visible at 4 months of age, we decided to evaluate the interobserver agreement at older ages as well.

Our first aim of the study was to investigate the interobserver agreement of the frequently used radiographic criteria for the diagnosis of hip laxity on Rottweiler dogs at 8 and 12 months of age. Furthermore, we aimed to compare the results reported in our previous article for the same Rottweilers by the same observers at 4 months of age [29] with our findings at 8 and 12 months of age. We hypothesized that the interobserver agreement increases with the age of the dogs; thus, we expected higher agreements regarding the older-aged dogs.

## 2. Materials and Methods

### 2.1. Approval and Study Design

The Institutional Ethics and Animal Welfare Committee of the University of Veterinary Medicine, Vienna discussed and approved this study in accordance with good scientific practice guidelines and national legislation (ETK-17/12/97/2015). We prospectively investigated twenty-nine purebred Rottweilers (five litters of puppies) of the Austrian Armed Forces at 8 and 12 months of age. All of the dogs had FCI pedigrees and each litter had different parents. The inclusion criteria included the absence of any clinical musculoskeletal diseases; thus, an orthopedic surgeon (B.V.) performed general clinical examinations prior to the study.

### 2.2. Interobserver Study

We based the interobserver study on the investigation of the eight radiographic parameters between the five observers. The radiographs were evaluated by two small animal orthopedic surgeons (Observers 1 and 2) and three veterinary radiologists (Observers 3, 4, and 5) in this study. Two of the observers (Observers 1 and 2) were certified CHD scrutineers; furthermore, Observers 1, 2, and 4 had performed radiographic measurements of the early diagnosis of CHD more than Observers 3 and 5, and they therefore had more experience in the early diagnosis of CHD. Each observer separately evaluated each parameter at each examination time (8 and 12 months of age).

### 2.3. Anesthesia

In this study, all of the dogs underwent general anesthesia and were premedicated with medetomidine (0.01–0.02 mg/kg, IV). The indication and maintenance of the anesthesia were performed with an intravenous injection of propofol (1–5 mg/kg and 0.2 mg/kg/min, respectively). Furthermore, atipamezol (0.05–0.1 mg/kg, IV) was used to antagonize the medetomidine after examinations.

### 2.4. Radiographic Examinations

Four different radiographic projections were used in this study to take the radiographs. The Norberg angle (NA), sclerosis of the cranial acetabular rim (SCAR), and location of the center of the femoral head (LCFH) were assessed in the ventrodorsal-view radiographs with extended legs [30]. We used ventrodorsal frog-leg view radiographs [31] to grade the degenerative joint disease (GDJD), and we took ventrodorsal-distraction-view radiographs [9] to measure the DI. We determined three other parameters, including the dorsal acetabular rim slope (DARS), center edge angle (CEA), and graded dorsal acetabular rim (GDAR) in the dorsal acetabular rim (DAR) view [32]. These eight values were divided into four quantitative (NA, DI, DARS, and CEA) and four qualitative (SCAR, LCFH, GDJD, and GDAR) parameters.

We took all the radiographs for this study at the Diagnostic Imaging of the University of Veterinary Medicine Vienna, and we exposed them with 75–96 kV and 9 mAs, with a film-focus distance of 90 cm, in a storage phosphor screen/cassette system (Kodak Carestream, Health Inc., Rochester, NY, USA). We digitally stored and evaluated all of the images (dicomPACS View Version 6.0.2, 457, Bodmin, England and Oehm und Rehbein, Rostock, Germany). An experienced technician anonymized the radiographs and their orders prior to the study.

#### 2.4.1. Norberg Angle (NA)

The NA is the angle between the straight line that connects the centers of the contralateral femoral heads in the ventrodorsal view and the line that connects the center of the femoral head at each side with the craniolateral border of the acetabulum [1]. An NA ≥ 105° is reported to be normal in most breeds, whereas smaller NAs may demonstrate luxated/subluxated femoral heads, or a poor covering of the femoral heads [12]. We measured the NA for each joint and documented it as a whole number.

#### 2.4.2. Sclerosis of Cranial Acetabular Rim (SCAR)

Based on its uniformity and thickness, in this study, we classified the SCAR as regular and thin sclerosis (normal), regular and thick sclerosis, or laterally increased thickness of sclerosis (abnormal).

#### 2.4.3. Location of Center of Femoral Head (LCFH)

According to its position in relation to the dorsal acetabular rim (medial, on, or lateral), we categorized the LCFH as medial to DAR (normal), superimposing DAR, 0–2 mm lateral to DAR, and more than 2 mm lateral to DAR.

#### 2.4.4. Grading of Degenerative Joint Disease (GDJD)

We graded the degenerative joint disease based on the joint space and severity of the degenerative changes. Based on these parameters, we graded the DJD of the coxofemoral joints as hips with congruent joint spaces and no osteophytes (normal), hips with incongruent joint spaces and no osteophytes (borderline), and hips with incongruent joint spaces with osteophytes including the Morgan Line (abnormal).

#### 2.4.5. Distraction Index (DI)

We used ventrodorsal-distraction-view radiographs [9] to measure the DI. We based the calculation of the DI on the division of the distance between the centers of the femoral head and acetabulum by the radius of the femoral head during distraction [16].

#### 2.4.6. Dorsal Acetabular Rim Slope (DARS)

We performed the measurement of the DARS in the DAR-view radiographs. To identify the DARS, we drew a straight line from the center of the femoral head to the most lateral point of the dorsal acetabular rim. Then, we drew a straight line tangent to this point. At the intersection of this straight line and the midsagittal axis of the pelvis/sacrum, we drew a perpendicular line. The DARS is the angle between these two straight lines, and it is ≤7.5° in healthy dogs [32].

#### 2.4.7. Center Edge Angle (CEA)

The CEA is the angle between the straight line that is drawn from the center of the femoral head parallel to the midsagittal axis of the pelvis/sacrum and the straight line that connects the center of the femoral head to the outer edge of the acetabular rim [33,34]. Healthy hips have a CEA > 12°, which demonstrates how much of the femoral head is covered by the acetabulum [34].

#### 2.4.8. Grading of Dorsal Acetabular Rim (GDAR)

We graded the dorsal acetabular rims according to their shapes and degenerative changes. We graded the hips with a triangular shape and no osteophytes as normal (Group 0). We graded the hips with a mild rounded DAR and no osteophytes (Group 1), rounded DAR with mild osteophytes (Group 2), truncated-shaped DAR, where the edges look cut off and with poor roofing (Group 3), and rounded-shape DAR with severe osteophytes (Group 4) as abnormal.

### 2.5. Statistical Analysis

The results were analyzed with IBM SPSS statistics version 27 in this study with a significance level of 0.05. We performed the general linear model (GLM) with multiple comparisons using Bonferroni’s alpha correction procedure to analyze the difference between the observers, as well as the examination times.

To assess the interobserver reliability of the quantitative parameters, we calculated the intraclass correlation coefficient (ICC). The ICC ranged from 0 (no agreement) to 1 (excellent agreement). We considered the values below 0.4 to be poor agreement, the values between 0.4 and 0.59 to be fair agreement, values between 0.6 and 0.74 to be good agreement, and values higher than 0.75 to be excellent agreement [35].

We calculated the interobserver reliability of the qualitative parameters using Cohen’s kappa coefficient (κ). The kappa ranged from 0 (no agreement) to 1 (excellent agreement). The parameters with a kappa below 0.20 indicated poor agreement, and the parameters between 0.21 and 0.40 indicated weak agreement. The parameters between 0.41 and 0.60 and between 0.61 and 0.80 indicated moderate and good agreements, respectively. The results with a kappa above 0.80 indicated excellent agreement.

In a few cases, Observer 5 interpreted the qualitative parameters as indefinable. Despite the low number of these cases, this might have had a negligible impact on the statistics.

## 3. Results

We evaluated twenty-nine purebred Rottweilers (58 hips) in this study. Twenty of the examined dogs were male (all intact) and eight of them were female (all intact). We performed the radiographic examinations at 8 and 12 months of age, with means and standard deviations (SDs) of 8.02 ± 0.4 months (range: 8.75–7.5 months) and 12.3 ± 0.25 months (range: 12.75–12 months), respectively. The means ± SDs of the body weights were 30.06 ± 2.36 kg (range: 38–25.1 kg) at 8 months and 34.53 ± 2.48 kg (range: 39–30.9 kg) at 12 months.

### 3.1. Quantitative Parameters

The means ± SDs of the NA at the first (8 months of age) and second (12 months of age) examination times were 105.90° ± 6.11° and 104.65° ± 6.8°, respectively. There were no significant differences in the recordings of the observers at any examination time.

The means ± SDs of the DI at 8 and 12 months of age were 0.21 ± 0.15 and 0.19 ± 0.14, respectively. We only recorded a significant difference between Observers 4 and 5 at 8 months of age, with a mean difference ± standard error (SE) of 0.08 ± 0.03 (*p* = 0.04), whereas we did not record any significant differences between the observers at 12 months of age.

The means ± SDs of the DARS at 8 and 12 months of age were 5.38° ± 3.87° and 5.96° ± 4.83°, respectively. We recorded significant differences between Observer 5 and all the other observers regarding the DARS at 8 months of age. We recorded the maximum difference between Observers 3 and 5, with a mean difference ± SE of 5.23° ± 0.66° (*p* < 0.001). At 12 months of age, we recorded six significant differences between the observers. We recorded the highest difference between Observers 3 and 5, with a mean difference ± SE of 7.13° ± 0.82° (*p* < 0.001).

The means ± SDs of the CEA at 8 and 12 months of age were 17.37° ± 4.95° and 15.92° ± 7.23°, respectively. In total, we observed three and four significant differences at 8 and 12 months of age, respectively, regarding the CEA. We recorded the highest difference at 8 months of age between Observers 2 and 5, with a mean difference ± SE of 4.40° ± 0.91° (*p* < 0.001), and we recorded the highest difference at 12 months of age between Observers 1 and 5, with a mean difference ± SE of 10.22° ± 1.23° (*p* < 0.001).

According to the results of the multiple comparisons between the examination times, including the examinations of the same parameters on the same animals at 4 months of age from our previous study [29], there was a significant difference between the measurements of the NA at 4 and 8 months, as well as at 4 and 12 months. We did not record any difference between 8 and 12 months of age regarding the measurements of the NA. We obtained the same results for the DI and DARS. The results reported for the CEA were different from those of the other three parameters. We did not record any significant difference between 4 and 8 months of age, or between 4 and 12 months of age, regarding the CEA, whereas we recorded a significant difference between 8 and 12 months of age. We present the multiple comparisons between the examination times of the quantitative values in Table 1.

We recorded excellent interobserver agreements for the quantitative parameters at 8 and 12 months of age. We present the results of the interobserver agreements of the quantitative values at 4 [29], 8, and 12 months of age in Table 2. Furthermore, in the Supplementary Materials, we also present the results of the general linear model and the Bonferroni’s alpha correction method (Figure S1).

**Figure 1:**
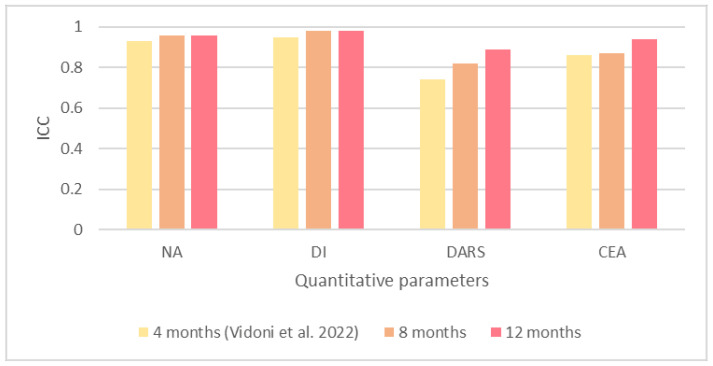
Illustration of intraclass correlation coefficient (ICC) of quantitative parameters at 4 [29], 8, and 12 months of age.

Based on the reported results, we recorded excellent agreements (ICC ≥ 0.75) for the NA, DI, and CEA at 4 [29], 8, and 12 months of age (Figure 1). We recorded a good agreement for the DARS at 4 months of age (ICC = 0.74) [29], while at 8 and 12 months of age, this agreement was improved to an excellent agreement (ICC ≥ 0.75).

### 3.2. Qualitative Parameters

#### 3.2.1. SCAR

Of the total SCAR measurements taken at 8 months of age, we diagnosed 71% as normal (regular or thin sclerosis), 12.4% as regular and thick sclerosis, and 16.6% as laterally increased thickness of sclerosis. At 12 months of age, we diagnosed 62.3% of the hips as normal (regular or thin sclerosis), 21.5% as regular and thick sclerosis, and 16.2% as laterally increased thickness of sclerosis. We present the results of the evaluation of the SCAR at 4 [29], 8, and 12 months of age in Table 3.

**Figure 2:**
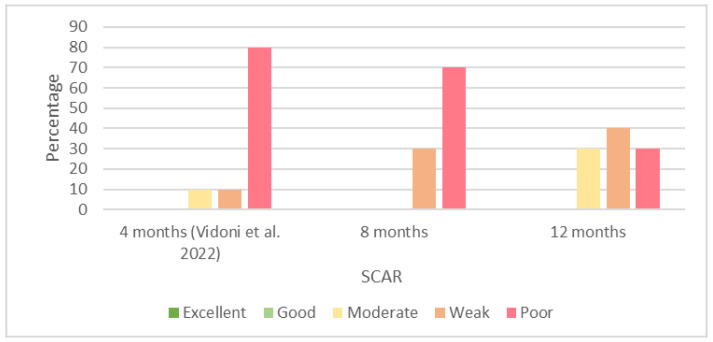
Percentages of results of interobserver agreements (Cohen’s kappa coefficient) for sclerosis of cranial acetabular rim (SCAR) at 4 [29], 8, and 12 months of age.

We did not record any excellent or good agreements for the SCAR measurements at 8 and 12 months of age. At 8 months of age, 30% of the agreements were weak, and 70% were poor. The results of the interobserver agreements at 12 months of age consisted of 30% moderate, 40% weak, and 30% poor agreements. The highest kappas recorded at 8 and 12 months of age were κ = 0.36 and κ = 0.39, respectively. We present the percentages of the results of the interobserver agreements of the SCAR at 4 [29], 8, and 12 months of age in Figure 2. Furthermore, we present the results of the Cohen’s kappa coefficient calculated for the qualitative parameters in the Supplementary Materials (Figure S2).

#### 3.2.2. LCFH

Of the total LCFH measurements at 8 months of age, we classified 74.4% of the femoral heads as medial to DAR, 13.5% as superimposing DAR, 7.3% as 0–2 mm lateral to DAR, and 4.5% as more than 2 mm lateral to DAR. At 12 months of age, we categorized 53.4% of the femoral heads as medial to DAR, 22.4% as superimposing DAR, 20.3% as 0–2 mm lateral to DAR, and 3.8% as more than 2 mm lateral to DAR. We present the results of the evaluation of the LCFH at 4 [29], 8, and 12 months of age in Table 4.

**Figure 3:**
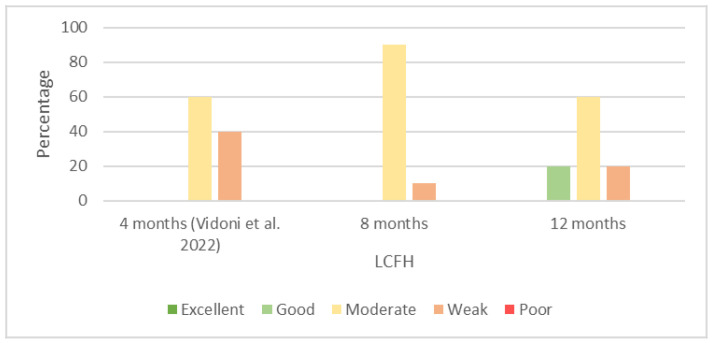
Percentages of results of interobserver agreements (Cohen’s kappa coefficient) for location of center of femoral head (LCFH) at 4 [29], 8, and 12 months of age.

Most of the recorded interobserver agreements regarding the LCFH at 8 months of age were moderate (90% of agreements), whereas we recorded 20% of the good agreements at 12 months of age. We recorded these good agreements between Observers 2 and 4 (κ = 0.61) and Observers 3 and 4 (κ = 0.68). We present the percentages of the results of the interobserver reliability of the LCFH at 4 [29], 8, and 12 months of age in Figure 3.

#### 3.2.3. GDJD

In total, at 8 months of age, we categorized 74.8% of the hips as hips with congruent joint spaces and no osteophytes, 24.5% of the hips as hips with incongruent joint spaces and no osteophytes, and 0.7% as hips with incongruent joint spaces with osteophytes. At 12 months of age, from the total of the GDJD measurements, we categorized 69.7% of the hips as hips with congruent joint spaces and no osteophytes, 27.6% of the hips as hips with incongruent joint spaces and no osteophytes, and 2.8% as hips with incongruent joint spaces with osteophytes. We present the results of the evaluation of the GDJD at 4 [29], 8, and 12 months of age in Table 5.

**Figure 4:**
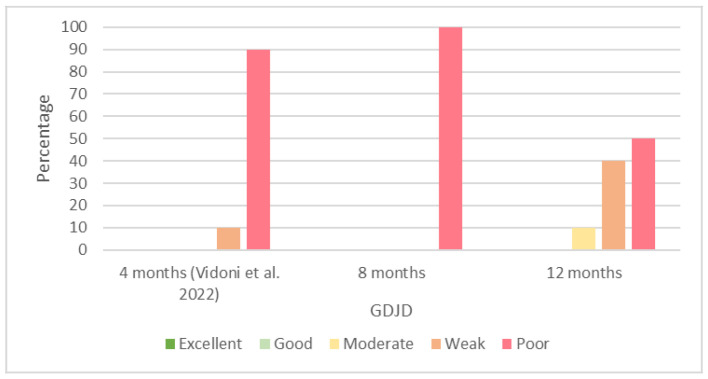
Percentages of results of interobserver agreements (Cohen’s kappa coefficient) for grading of degenerative joint disease (GDJD) at 4 [29], 8, and 12 months of age.

We did not record any good or excellent interobserver agreements for the GDJD at 8 and 12 months of age. We present the percentages of the results of the interobserver agreements of the GDJD at 4 [29], 8, and 12 months of age in Figure 4.

#### 3.2.4. GDAR

From the total of the measurements at 8 months of age, we diagnosed 62.1% with a triangular shape and no osteophytes, 32.4% with a mild rounded DAR and no osteophytes, 3.8% with a rounded DAR with mild osteophytes, 1.4% with the truncated shape of the DAR, where the edge looks cut off or dabbed and there is less roofing, and 0.3% as the rounded shape of the DAR with severe osteophytes. At 12 months of age, we diagnosed 55% with a triangular shape and no osteophytes, 34.6% with a mild rounded DAR and no osteophytes, 5.7% with a rounded DAR with mild osteophytes, 2.9% with the truncated shape of the DAR, where the edge looks cut off or dabbed and there is less roofing, and 1.8% with the rounded shape of the DAR with severe osteophytes. We present the results of the evaluation of the GDAR at 4 [29], 8, and 12 months of age in Table 6.

**Figure 5:**
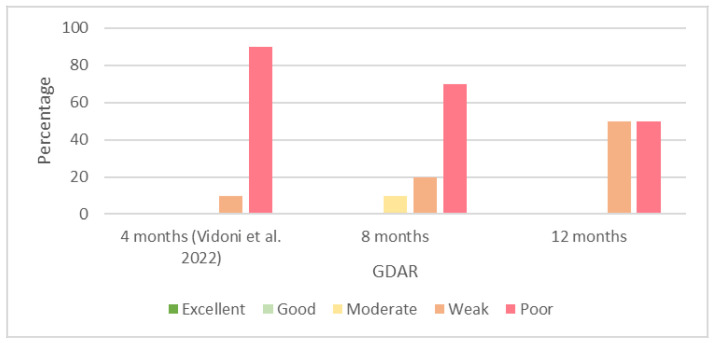
Percentages of results of interobserver agreements (Cohen’s kappa coefficient) for grading of dorsal acetabular rim (GDAR) at 4 [29], 8, and 12 months of age.

Most of the interobserver agreements recorded for the GDAR at 8 and 12 months of age were poor or weak. We did not observe any excellent or good agreements for the GDAR. We present the percentages of the results of the interobserver reliability of the GDAR at 4 [29], 8, and 12 months of age in Figure 5.

## 4. Discussion

In this study, our aim was to investigate the interobserver reliability of the eight most commonly used radiographic criteria, including four quantitative (NA, DI, DARS, and CEA) and four qualitative (SCAR, LCFH, GDJD, and GDAR) parameters, for the diagnosis of CHD in purebred Rottweilers at 8 and 12 months of age, and to compare the recorded results with the results that we previously reported for the dogs at 4 months of age [29]. We hypothesized that the interobserver agreement would increase with the age of the dogs.

The results recorded for the quantitative parameters confirmed our hypothesis, and especially in comparison with those at 4 months of age that we previously reported [29]. Except in a few cases, we did not observe any remarkable high agreements regarding the qualitative values. Most of the interobserver agreements of the qualitative values were improved from poor/weak to weak/moderate agreements in the older ages, and except for a few cases, we did not observe any good or excellent agreements. Therefore, our hypothesis was not confirmed for the qualitative values in this study.

According to the results of our study, there were excellent interobserver agreements for all the quantitative values at 8 and 12 months of age. The ICC values of the NA and DI were the same at both ages (NA = 0.96 and DI = 0.98), whereas the ICC values recorded for the DARS and CEA, despite being excellent at both ages, slightly increased at 12 months of age. These results are more prominent when we compare them with results for the same dogs at 4 months of age by the same observers, which we reported in our previous study [29].

We observed an excellent interobserver agreement for the NA at 4 months of age [29], and this agreement even increased at 8 months of age and remained the same at 12 months of age. Similar to the NA, we recorded an excellent interobserver agreement for the DI at 4 months of age [29], which was increased at 8 months of age and remained the same at 12 months.

We recorded good interobserver agreement for the DARS at 4 months of age [29], which was improved to excellent agreement at 8 months of age, and even increased at 12 months of age, which was the largest improvement among the quantitative values. The interobserver agreement reported for the CEA at 4 months of age was excellent [29]. This excellent agreement was slightly increased at 8 months of age, and thereafter at 12 months of age.

According to the multiple comparisons between the mean values of the NA, DI, and DARS, there were no significant differences between the measurements at 8 and 12 months of age, whereas the same values between 4 [29] and 8 months of age, as well as between 4 [29] and 12 months of age, were significantly different (*p* < 0.001). Despite these parameters, the results reported for the CEA were completely different. We did not record any significant differences between the measurements of the CEA at 4 [29] and 8 months of age, or between 4 [29] and 12 months of age; however, the measurements between 8 and 12 months of age significantly differed (*p* = 0.013). Due to these findings, on the one hand, we can conclude that the evaluation of the NA, DI, and DARS at 8 months of age did not differ from those at 12 months of age, and we can expect approximately the same results; on the other hand, the measurements of the CEA differed in the older ages, and we should expect even higher interobserver agreements at 12 months of age.

One of the reasons for the increased interobserver agreement of the DARS and CEA for the older ages might be the skeletal maturity of the dogs. They reach their primary skeletal growth and muscle consistency at 4 months of age, and from this age, veterinarians can perform predictive radiographic and orthopedic examinations, such as the Ortolani maneuver [36,37,38]. At 8 months of age, dogs reach the main peak of their skeletal maturity, and it is mostly completed by 12 months of age (18 months in some breeds). Due to the difficulty of the detection of the center of the femoral head, craniolateral edge of the acetabulum, and dorsal acetabular rim on DAR-view radiographs of juvenile dogs, the interobserver agreements increased with the age of the dogs, as the observers could better detect them at the older ages.

Based on the multiple comparisons between the mean values of each quantitative parameter at 4 [29], 8, and 12 months of age, there were no significant differences for the NA measurements, and we did not report any significant differences for the DI at 4 months of age [29]. We only recorded one significant difference at 8 months of age and two significant differences at 12 months of age for the DI measurements. According to the comparisons between the DARS measurements on all the examination dates, the number of significant differences between the observers was similar at 4 (seven differences) [29] and 12 (six differences) months of age, whereas this number was slightly lower at 8 months of age (four differences). We recorded most of these differences between Observer 3 (radiologist), Observer 5 (radiologist), and the other observers. The significant difference recorded for the CEA increased from one difference at 4 months of age [29] to three differences at 8 months of age and four differences at 12 months of age. These differences at 4 [29] and 8 months of age regarding the CEA were not specific to a particular observer group or person, and they equally existed between the surgeons and radiologists. However, at 12 months of age, all the differences that we recorded were between Observer 1 (surgeon) and the other observers. In general, Observers 1, 2, and 4 had the lowest number of differences between each other in our previous [29] and the current study. The similar results between these observers might be because of the daily repetition of these measurements, or they could be due to the experience of the observers in the early diagnosis of hip laxity.

The interobserver agreements recorded for the qualitative values were mostly from poor to moderate at both the examination times in this study. Most of the interobserver agreements recorded for the SCAR at 8 months of age were poor, similar to those recorded at 4 months of age [29]; however, these poor agreements were reduced at 12 months of age, when we recorded more moderate agreements. In comparison with our previous study [29], the number of poor agreements was reduced from 80% at 4 months of age to 30% at 12 months of age, while we did not observe any excellent or good agreements for the older ages. Thus, despite the negligible improvement in the interobserver agreements, the increasing age did not increase the interobserver agreement of the SCAR evaluations.

We recorded a high number of moderate interobserver agreements at 8 and 12 months of age regarding the LCFH. The numbers of moderate agreements at 4 [29] and 12 months of age were the same (60%). The difference between these two ages was in the reduced weak and increased good agreements at 12 months of age.

Despite a high number of poor interobserver agreements of the GDJD at 4 [29] and 8 months of age, we observed a minimal improvement at 12 months of age, and the poor results were reduced from 100% at 8 months to 50% at 12 months, while we did not observe any good agreements at all.

Similar to the GDJD, we recorded a high number of poor results for the GDAR. The number of poor agreements at 4 months of age [29] was 90%; however, the number decreased to 70% and 50% at 8 and 12 months of age, respectively. We did not observe any excellent or good agreements regarding the GDAR at any age in our study.

As reported in previous studies, qualitative values are more relative to the observers than quantitative values, and different observers might have different opinions for the same case; thus, we can expect a broad range of results [29,39]. The standardization of the film-reading process might increase the interobserver agreements and help the veterinarian to reduce the number of false-negative or false-positive results [39]. False-negative or -positive results mostly occur on borderline hips because their detection is more difficult than the detection of healthy or severely affected hips. In the case of a false-negative diagnosis, dysplastic dogs will be included in breeding programs, which may contribute to slow progress in terms of reducing the CHD incidence, despite screening programs [39,40]. False-positive results will exclude nondysplastic dogs from breeding programs, which reduces the population of nondysplastic dogs and also may increase the risk of other genetic disorders due to the reduction in the genetic variation in dogs [39]. The main reasons for the diagnosis of false-negative or false-positive hips are the low interobserver agreement of the screening programs and the late onset of the degenerative disease [40]. Using screening programs with high interobserver agreements will decrease the number of false-negative or false-positive results.

In a study performed by Verhoeven et al. [39], the authors report low interobserver agreements for the diagnosis of CHD with the FCI scoring method. Paster et al. [41], Saunders et al. [42], Smith et al. [43], and Fortrie et al. [44] report the same results for subjective hip scoring with ventrodorsal hip-extended radiographs. These studies support our findings regarding the low interobserver agreements for the qualitative values.

Despite the low interobserver agreements for the qualitative parameters, researchers have reported good agreements in the literature for most of the quantitative values. In a study performed by Broeckx et al. [45], despite a substantial measurement bias between the observers regarding the NA, the authors observed good interobserver agreements regarding the DI and laxity index. Bertal et al. [46] confirmed these findings. In another study performed by Klever et al. [47], the authors recorded high interobserver agreements for the measurements of the NA and DI between novice and experienced observers. These findings support our findings regarding the high interobserver agreements for the quantitative values.

The experience of the examiners is an important topic in intra- and inter-observer studies. In previous studies, researchers report various results regarding the effect of the experience of the observers on the intra- and inter-observer agreements. Studies in which researchers investigated the qualitative or subjective parameters, such as the DJD, had better interobserver agreements between the experienced observers than the novice observers [39]; in other studies, in which the researchers evaluated the quantitative values, such as the DI or NA, they report no significant correlation between the degree of the experience of the observers and the interobserver agreements. Therefore, the experience of the observers, or even the self-learning of each observer, did not increase the intra- and inter-observer agreements for the quantitative parameters in these studies [46,47].

In our study, we did not record any significant differences between the observers regarding the NA and DI (except in a few cases) measurements, which confirms our previous studies [46,47]; however, we did record significant differences between the observers regarding the DARS and CEA measurements at 8 and 12 months of age. Observers 3 and 5, despite being experts in diagnostic imaging, had performed these measurements less than the other three observers had; thus, they had less experience in the early diagnosis of hip laxity. Most of the differences recorded for the CEA at 12 months of age were between one of the experienced observers (Observer 1) and the others; thus, we cannot consider the experience of the observers, despite being an important aspect, as an absolute factor because observers with a lot of experience might also produce different results. We only recorded these differences for the complex measurements (the DARS and CEA) of the quantitative values. Considering that the complexity of the structures can decrease the intra- and inter-observer agreements [48], the recommendation is to use simple parameters, such as the DI or NA, which had high intra- and inter-observer agreements, or to use a combination of different methods, such as the total score reported by Merca et al. (2020) [49] together with other methods, or other modified methods, such as the modified FCI reported by Mostafa et al. (2022) [28]. However, we recommend reducing the number of observers to reduce the interobserver variability [46,50]. Our findings on the effect of the experience of the observers and the qualitative values were contrary to previous studies, and we recorded the same results (poor–moderate agreements) between the novice and experienced observers. We require further investigations to assess the relationship between the degree of the experience of the observers and the different parameters, and especially for the subjective parameters.

The quality of the radiographs and positioning errors are important issues in radiographic studies. Experienced technicians took all of the radiographs included in this study, and an experienced radiologist controlled the process to meet the inclusion criteria. Having high-quality radiographs facilitates the radiographic evaluation and film-reading process; however, it does not influence the agreements, as all the observers receive and evaluate the same radiographs [51].

In this study, we evaluated the interobserver variability, which provided information on the reproducibility of the method on 8- and 12-month-old Rottweilers. In the future, researchers could use the intraobserver variability at the same ages to investigate the repeatability of the methods. Furthermore, an assessment of only one specific breed may not be expandable to other dog breeds; therefore, we recommend investigations into the intra- and inter-observer agreements for other dog breeds as well.

According to Observer 5, some quantitative parameters were indefinable in our study. Potentially negligible effects on the statistical findings could have been caused by these missing data.

## 5. Conclusions

We recorded from excellent to good interobserver agreements for the NA, DI, DARS, and CEA in our study at 8 and 12 months of age, whereas we recorded from poor to moderate agreements for the SCAR, LCFH, GDJD, and GDAR at both ages. The data presented in this study do not help us to understand the extent to which the level of the observer’s experience influences the accuracy of the diagnosis; however, the NA, DI, DARS, and CEA were neither reporter-experienced nor dog-age-dependent. Thus, we could not draw a firm conclusion in this study as to the correlation between the level of experience of the observers and the interobserver agreements.

## Figures and Tables

**Table 1:** Multiple comparisons of measurements of quantitative values with mean differences and standard errors at 4 [29], 8, and 12 months of age with Bonferroni’s alpha correction.

Parameter	Age (Months) (Mean ± SD *)		Mean Difference	Standard Error	Significance
NA (°)	8 (105.90 ± 6.11)	4 ** (101.80 ± 7.1)	4.10	0.57	0.000
		12 (104.65 ± 6.8)	1.26	0.57	0.083
	12	4 **	2.84	0.58	0.000
DI (mm)	8 (0.21 ± 0.15)	4 ** (0.34 ± 0.2)	−0.13	0.01	0.000
		12 (0.19 ± 0.14)	0.01	0.01	0.837
	12	4 **	−0.15	0.01	0.000
DARS (°)	8 (5.38 ± 3.87)	4 ** (7.84 ± 5)	−2.46	0.40	0.000
		12 (5.96 ± 4.83)	−0.58	0.40	0.442
	12	4 **	−1.88	0.40	0.000
CEA (°)	8 (17.37 ± 4.95)	4 ** (16.2 ± 5.5)	1.06	0.51	0.111
		12 (15.92 ± 7.23)	1.45	0.51	0.013
	12	4 **	−0.38	0.51	1.000

* Standard deviation; ** values from Vidoni et al. (2022) [29].

**Table 2:** Intraclass correlation coefficient (ICC) for interobserver variability between observers.

Parameter	Age (Months)	ICC	Significance
NA	4 *	0.93	*p* < 0.001
	8	0.96	*p* < 0.001
	12	0.96	*p* < 0.001
DI	4 *	0.95	*p* < 0.001
	8	0.98	*p* < 0.001
	12	0.98	*p* < 0.001
DARS	4 *	0.74	*p* < 0.001
	8	0.82	*p* < 0.001
	12	0.89	*p* < 0.001
CEA	4 *	0.86	*p* < 0.001
	8	0.87	*p* < 0.001
	12	0.94	*p* < 0.001

* Values from Vidoni et al. (2022) [29].

**Table 3:** Percentages (%) of sclerosis of cranial acetabular rim (SCAR) evaluations by observers at 4 [29], 8, and 12 months of age.

Observer	Regular or Thin Sclerosis			Regular and Thick Sclerosis			Laterally Increased Thickness of Sclerosis		
	4 M **	8 M	12 M	4 M **	8 M	12 M	4 M **	8 M	12 M
1 (Surgeon) *	100.0	96.6	82.8	0	3.4	15.5	0	0	1.7
2 (Surgeon)	76.8	51.7	46.6	23.2	20.7	32.8	0	27.6	20.7
3 (Radiologist)	68.5	72.4	67.2	16.7	17.2	19	14.8	10.3	13.8
4 (Radiologist) *	96.4	63.8	63.8	3.6	0	8.6	0	36.2	27.6
5 (Radiologist)	70.0	71	62.3	30.0	12.4	21.5	0	16.6	16.2
Total	84.1	71	62.3	12.6	12.4	21.5	3.3	16.6	16.2

* Certified CHD scrutineer; ** values from Vidoni et al. (2022) [29]. 4 M: 4 months of age; 8 M: 8 months of age; 12 M: 12 months of age.

**Table 4:** Percentages (%) of evaluations of location of center of femoral head (LCFH) by observers at 4 [29], 8, and 12 months of age.

Observer	Medial to DAR ^†^			Superimposing DAR			0–2 mm Lateral to DAR			More than 2 mm Lateral to DAR		
	4 M **	8 M	12 M	4 M **	8 M	12 M	4 M **	8 M	12 M	4 M **	8 M	12 M
1 (Surgeon) *	53.7	77.6	55.2	37.0	17.2	31	7.4	1.7	13.8	1.9	3.4	0
2 (Surgeon)	57.1	72.4	44.8	19.6	10.3	19	21.4	12.1	31	1.8	5.2	5.2
3 (Radiologist)	44.6	79.3	63.8	25.0	3.4	12.1	23.2	12.1	20.7	7.1	5.2	3.4
4 (Radiologist) *	41.2	73.7	51.7	35.3	12.3	22.4	21.6	10.5	20.7	2.0	3.5	5.2
5 (Radiologist)	49.0	70.7	51.7	29.4	24.1	27.6	19.6	0	15.5	2.0	5.2	5.2
Total	49.3	74.7	53.4	29.1	13.5	22.4	18.7	7.3	20.3	3.0	4.5	3.8

^†^ DAR: dorsal acetabular rim; * certified CHD scrutineer; ** values from Vidoni et al. (2022) [29]. 4 M: 4 months of age; 8 M: 8 months of age; 12 M: 12 months of age.

**Table 5:** Percentages (%) of evaluations of grading of degenerative joint disease (GDJD) by observers at 4 [29], 8, and 12 months of age.

Observer	Congruent Joint Space and No Osteophytes			Incongruent Joint Space and No Osteophytes			Incongruent Joint Space with Osteophytes		
	4 M **	8 M	12 M	4 M **	8 M	12 M	4 M **	8 M	12 M
1 (Surgeon) *	88.9	93.1	70.7	11.1	5.2	25.9	0	1.7	3.4
2 (Surgeon)	55.4	44.8	41.4	44.6	55.2	55.2	0	0	3.4
3 (Radiologist)	44.6	82.8	84.5	44.6	17.2	13.8	10.7	0	1.7
4 (Radiologist) *	76.8	60.3	55.2	21.4	39.7	43.1	1.8	0	1.7
5 (Radiologist)	75.9	94.4	96.6	11.1	3.7	0	13.0	1.9	3.4
Total	68.1	74.8	69.7	26.8	24.5	27.6	5.1	0.7	2.8

* Certified CHD scrutineer; ** values from Vidoni et al. (2022) [29]. 4 M: 4 months of age; 8 M: 8 months of age; 12 M: 12 months of age.

**Table 6:** Percentages (%) of evaluations of grading of dorsal acetabular rim (GDAR) by observers at 4 [29], 8, and 12 months of age.

Observer	Triangular Shape and No Osteophytes			Mildly Rounded DAR ^†^ and No Osteophytes			Rounded DAR with Mild Osteophytes			Truncated Shape of the DAR with Laxity and Less Roofing			Rounded Shape of the DAR with Severe Osteophytes		
	4 M **	8 M	12 M	4 M **	8 M	12 M	4 M **	8 M	12 M	4 M **	8 M	12 M	4 M **	8 M	12 M
1 (Surgeon) *	79.6	94.8	67.2	18.5	1.7	29.3	0	1.7	0	1.9	0	1.7	0	1.7	1.7
2 (Surgeon)	12.5	44.8	33.9	69.6	51.7	58.9	17.9	1.7	0	0	5.4	5.4	0	0	1.8
3 (Radiologist)	1.8	56.9	39.3	41.1	31	37.5	35.7	8.6	17.9	16.1	3.4	3.6	5.4	0	1.8
4 (Radiologist) *	37.5	75.9	83.9	41.1	19	12.5	16.1	5.2	1.8	5.4	0	0	0	0	1.8
5 (Radiologist)	28.3	37.9	50	32.1	58.6	35.2	26.4	1.7	9.3	7.5	1.7	3.7	5.7	0	1.9
Total	31.6	62.1	55	40.7	32.4	34.6	19.3	3.8	5.7	6.2	1.4	2.9	2.2	0.3	1.8

^†^ DAR: dorsal acetabular rim; * certified CHD scrutineer; ** values from Vidoni et al. (2022) [29]. 4 M: 4 months of age; 8 M: 8 months of age; 12 M: 12 months of age.

## Data Availability

The data presented in this study are available in the Supplementary Materials.

## Supplementary Materials

The following supporting information can be downloaded at: https://www.mdpi.com/article/10.3390/ani13020231/s1. Figure S1: Result of general linear model and Bonferroni’s alpha correction procedure: results of multiple comparisons between 4, 8, and 12 months of age. Figure S2: Results of Cohen’s kappa coefficient calculated for qualitative parameters at 8 and 12 months of age.
