# Supplementary material for: Radiographic Diagnosis of Hip Laxity in Rottweilers: Interobserver Agreement at Eight- and Twelve-Months of Age
Source: Animals (Basel). 2023 Jan 8;13(2):231. doi: 10.3390/ani13020231 (PMC9855059; doi:10.3390/ani13020231)
Supplement: Supplementary file 1 [file animals-13-00231-s001.zip › animals-2109789-supplementary.pdf]

Supplementary Materials

Figure S1. Result of general linear model and Bonferroni's alpha correction procedure: results of multiple comparisons between 4, 8, and 12 months of age.

Multiple Comparisons

Bonferroni

Dependent Variable	(I) Examination_Date	(J) Examination_Date	Mean Difference (I-J)	Std. Error	Sig.	95% Confidence Interval	
						Lower Bound	Upper Bound
NA	4 Month Old	8 Month Old	-4,1032*	,57140	,000	-5,4740	-2,7324
		12 Month Old	-2,8464*	,57761	,000	-4,2321	-1,4608
	8 Month Old	4 Month Old	4,1032*	,57140	,000	2,7324	5,4740
		12 Month Old	1,2568	,56975	,083	-,1101	2,6236
	12 Month Old	4 Month Old	2,8464*	,57761	,000	1,4608	4,2321
		8 Month Old	-1,2568	,56975	,083	-2,6236	,1101
DI	4 Month Old	8 Month Old	,1352*	,01305	,000	,1038	,1665
		12 Month Old	,1493*	,01320	,000	,1176	,1809
	8 Month Old	4 Month Old	-,1352*	,01305	,000	-,1665	-,1038
		12 Month Old	,0141	,01302	,837	-,0171	,0453
	12 Month Old	4 Month Old	-,1493*	,01320	,000	-,1809	-,1176
		8 Month Old	-,0141	,01302	,837	-,0453	,0171
DAR_Slop	4 Month Old	8 Month Old	2,4590*	,39939	,000	1,5009	3,4171
		12 Month Old	1,8815*	,40373	,000	,9129	2,8500
	8 Month Old	4 Month Old	-2,4590*	,39939	,000	-3,4171	-1,5009
		12 Month Old	-,5775	,39823	,442	-1,5329	,3778
	12 Month Old	4 Month Old	-1,8815*	,40373	,000	-2,8500	-,9129
		8 Month Old	,5775	,39823	,442	-,3778	1,5329
CEA	4 Month Old	8 Month Old	-1,0621	,50876	,111	-2,2826	,1584
		12 Month Old	,3844	,51429	1,000	-,8494	1,6182
	8 Month Old	4 Month Old	1,0621	,50876	,111	-,1584	2,2826
		12 Month Old	1,4465*	,50729	,013	,2295	2,6635
	12 Month Old	4 Month Old	-,3844	,51429	1,000	-1,6182	,8494
		8 Month Old	-1,4465*	,50729	,013	-2,6635	-,2295

Based on observed means.
The error term is Mean Square(Error) = 35,111.

*. The mean difference is significant at the ,05 level.

Figure S2. Results of Cohen's kappa coefficient calculated for qualitative parameters at 8 and 12 months of age.

The inter-observer reliability of the qualitative parameters was calculated using Cohen's kappa coefficient (κ). The kappa ranges from 0 (no agreement) to 1 (excellent agreement). The parameters with a kappa below 0.20 indicate poor agreement, and the parameters between 0.21 and 0.40 indicate weak agreement. The parameters between 0.41 and 0.60, and between 0.61 and 0.80 indicate moderate and good agreements, respectively. Results with a kappa above 0.80 indicate excellent agreement.

SCAR eight months of age

	OBSERVER 1	OBSERVER 2	OBSERVER 3	OBSERVER 4	OBSERVER 5
OBSERVER 1		0.09	0.06	0.06	0.17
OBSERVER 2	0.09		0.05	0.36	0.30
OBSERVER 3	0.06	0.05		0.12	0.11
OBSERVER 4	0.06	0.36	0.12		0.25
OBSERVER 5	0.17	0.30	0.11	0.25	

SCAR 12 months of age

	OBSERVER 1	OBSERVER 2	OBSERVER 3	OBSERVER 4	OBSERVER 5
OBSERVER 1		0.09	0.41	0.28	0.14
OBSERVER 2	0.09		0.27	0.26	0.42
OBSERVER 3	0.41	0.27		0.43	0.39
OBSERVER 4	0.28	0.26	0.43		0.39
OBSERVER 5	0.14	0.42	0.39	0.39	

LCFH eight months of age

	OBSERVER 1	OBSERVER 2	OBSERVER 3	OBSERVER 4	OBSERVER 5
OBSERVER 1		0.46	0.4	0.54	0.53
OBSERVER 2	0.46		0.57	0.48	0.48
OBSERVER 3	0.42	0.57		0.57	0.40
OBSERVER 4	0.54	0.48	0.57		0.43
OBSERVER 5	0.53	0.48	0.40	0.43	

LCFH 12 months of age

	OBSERVER 1	OBSERVER 2	OBSERVER 3	OBSERVER 4	OBSERVER 5
OBSERVER 1		0.26	0.56	0.50	0.60
OBSERVER 2	0.26		0.45	0.61	0.29
OBSERVER 3	0.56	0.45		0.68	0.51
OBSERVER 4	0.50	0.61	0.68		0.48
OBSERVER 5	0.60	0.29	0.51	0.48	

GDJD eight months of age

	OBSERVER 1	OBSERVER 2	OBSERVER 3	OBSERVER 4	OBSERVER 5
OBSERVER 1		0.03	0.14	0.06	0.10
OBSERVER 2	0.03		0.10	0.06	0.08
OBSERVER 3	0.14	0.10		0.18	0.09
OBSERVER 4	0.06	0.06	0.18		0.09
OBSERVER 5	0.10	0.08	0.09	0.09	

GDJD 12 months of age

	OBSERVER 1	OBSERVER 2	OBSERVER 3	OBSERVER 4	OBSERVER 5
OBSERVER 1		0.30	0.39	0.51	0.18
OBSERVER 2	0.30		0.16	0.06	0.08
OBSERVER 3	0.39	0.16		0.24	0.25
OBSERVER 4	0.51	0.06	0.24		0.08
OBSERVER 5	0.18	0.08	0.25	0.08	

GDAR eight months of age

	OBSERVER 1	OBSERVER 2	OBSERVER 3	OBSERVER 4	OBSERVER 5
OBSERVER 1		0.09	0.09	0.19	0.07
OBSERVER 2	0.09		0.29	0.2	0.41
OBSERVER 3	0.09	0.29		0.01	0.25
OBSERVER 4	0.19	0.2	0.01		0.2
OBSERVER 5	0.07	0.41	0.25	0.2	

GDAR 12 months of age

	OBSERVER 1	OBSERVER 2	OBSERVER 3	OBSERVER 4	OBSERVER 5
OBSERVER 1		0.11	0.26	0.01	0.39
OBSERVER 2	0.11		0.28	0.19	0.21
OBSERVER 3	0.26	0.28		0.16	0.39
OBSERVER 4	0.01	0.19	0.16		0.12
OBSERVER 5	0.39	0.21	0.39	0.12	
